# Using the Theoretical Domains Framework to Identify Barriers and Enablers to Implementing a Virtual Tertiary–Regional Telemedicine Rounding and Consultation for Kids (TRaC-K) Model: Qualitative Study

**DOI:** 10.2196/28610

**Published:** 2021-12-22

**Authors:** Sumedh Bele, Christine Cassidy, Janet Curran, David W Johnson, J A Michelle Bailey

**Affiliations:** 1 Department of Community Health Sciences Cumming School of Medicine University of Calgary Calgary, AB Canada; 2 Department of Pediatrics Cumming School of Medicine University of Calgary Calgary, AB Canada; 3 School of Nursing Dalhousie University Halifax, NS Canada; 4 Department of Pediatrics Alberta Health Services Calgary, AB Canada

**Keywords:** telemedicine, eHealth, pediatric care, inpatient, regional, rural, Canada, Theoretical Domains Framework, qualitative

## Abstract

**Background:**

Inequities in access to health services are a global concern and a concern for Canadian populations living in rural areas. Rural children hospitalized at tertiary children’s hospitals have higher rates of medical complexity and experience more expensive hospitalizations and more frequent readmissions. The 2 tertiary pediatric hospitals in Alberta, Canada, have already been operating above capacity, but the pediatric beds at regional hospitals are underused. Such imbalance could lead to poor patient safety and increased readmission risk at tertiary pediatric hospitals and diminish the clinical exposure of regional pediatric health care providers, erode their confidence, and compel health systems to further reduce the capacity at regional sites. A *Telemedicine Rounding and Consultation for Kids* (TRaC-K) model was proposed to enable health care providers at Alberta Children’s Hospital to partner with their counterparts at Medicine Hat Regional Hospital to provide inpatient clinical care for pediatric patients who would otherwise have to travel or be transferred to the tertiary site.

**Objective:**

The aim of this study is to identify perceived barriers and enablers to implementing the TRaC-K model.

**Methods:**

This study was guided by the Theoretical Domains Framework (TDF) and used qualitative methods. We collected qualitative data from 42 participants from tertiary and regional hospitals through 31 semistructured interviews and 2 focus groups. These data were thematically analyzed to identify major subthemes within each TDF domain. These subthemes were further aggregated and categorized into barriers or enablers to implementing the TRaC-K model and were tabulated separately.

**Results:**

Our study identified 31 subthemes in 14 TDF domains, ranging from administrative issues to specific clinical conditions. We were able to merge these subthemes into larger themes and categorize them into 4 barriers and 4 enablers. Our findings showed that the barriers were lack of awareness of telemedicine, skills to provide virtual clinical care, unclear processes and resources to support TRaC-K, and concerns about clear roles and responsibilities. The enablers were health care providers’ motivation to provide care closer to home, supporting system resource stewardship, site and practice compatibility, and motivation to strengthen tertiary–regional relationships.

**Conclusions:**

This systematic inquiry into the perceived barriers and enablers to the implementation of TRaC-K helped us to gain insights from various health care providers’ and family members’ perspectives. We will use these findings to design interventions to overcome the identified barriers and harness the enablers to encourage successful implementation of TRaC-K. These findings will inform the implementation of telemedicine-based interventions in pediatric settings in other parts of Canada and beyond.

**International Registered Report Identifier (IRRID):**

RR2-10.1186/s12913-018-3859-2

## Introduction

### Background

The World Health Organization considers “providing equitable access to people-centered care” as one of the key components of a well-functioning health system [1]. In Canada, almost one-fifth of the population (18%) lives in rural communities, but they are served by only 8% of the physicians [2]. Evidence suggests that rural children hospitalized at tertiary children’s hospitals have higher rates of medical complexity and experience more expensive hospitalizations and more frequent readmissions [3]. Although inequitable access to health services is a global concern, it is paramount for provinces in Canada to efficiently allocate health resources to reduce health care spending and provide equitable access to pediatric patients in nonmetropolitan and rural communities.

Alberta Children’s Hospital (ACH) and Stollery Children’s Hospital are the 2 tertiary pediatric hospitals in Alberta. Several regional hospitals throughout the province also have dedicated pediatric beds. Medicine Hat Regional Hospital (MHRH) is one such regional hospital located 300 km southeast of Calgary. Alberta Health Services (AHS) is the single health authority for Alberta province and is the largest such organization in Canada. Both ACH and MHRH are part of AHS. In 2015-16, ACH operated at 95.1% of its bed capacity half of the year and at more than 100% during the rest of the year. During the same year, MHRH showed only 57% occupancy rates. Furthermore, in that same year, 28% of the total pediatric inpatient days of stay at ACH were used by 19% of the pediatric patients from the MHRH catchment area. High bed occupancy is not only associated with poor patient safety, high mortality rates, and increased readmission risk, but it also increases the stress of health care providers [4,5]. In contrast, low use of beds in regional hospitals may diminish the clinical exposure of regional pediatric health care providers, erode their confidence, and compel health systems to further reduce the capacity at regional sites, which hampers access to specialized care for rural patients [6].

Telemedicine is a way to address tertiary–rural imbalances in health care delivery. The World Health Organization defines telemedicine as “healing at a distance” through the use of information and communication technologies (ICTs) [7]. The use of telemedicine enables health care systems to connect patients with health care providers in underserved areas, save time and travel expenses for the patients and their families, increase efficiency, and improve quality of care while supporting health care providers at rural, regional, or community health care sites [8,9]. Telemedicine is an umbrella term that incorporates the use of various forms of ICTs, including but not limited to telephone, electronic messaging, SMS text messaging, mobile apps, and audiovisual tools to provide clinical care. Among these various ICT applications, audiovisual systems are the most promising because they provide an opportunity for real-time audiovisual communication between 2 sites, which facilitates better understanding of a patient’s clinical condition, more accurate diagnosis and clinical management, and enhanced communication between personnel at both sites [10]. A growing body of evidence suggests that telemedicine has the potential to bridge geographical barriers to providing clinical care, ranging from diagnosis and active management to maintaining continuity of care for pediatric patients [8]. The educational applications of telemedicine lie in its ability to connect tertiary teaching hospitals with community or rural practices, allowing bidirectional flow of information [11]. Telemedicine also enhances personal relationships between rural pediatricians and subspecialists [12].

### Telemedicine-Facilitated Model for Inpatient Pediatric Clinical Care

**Figure 1:**
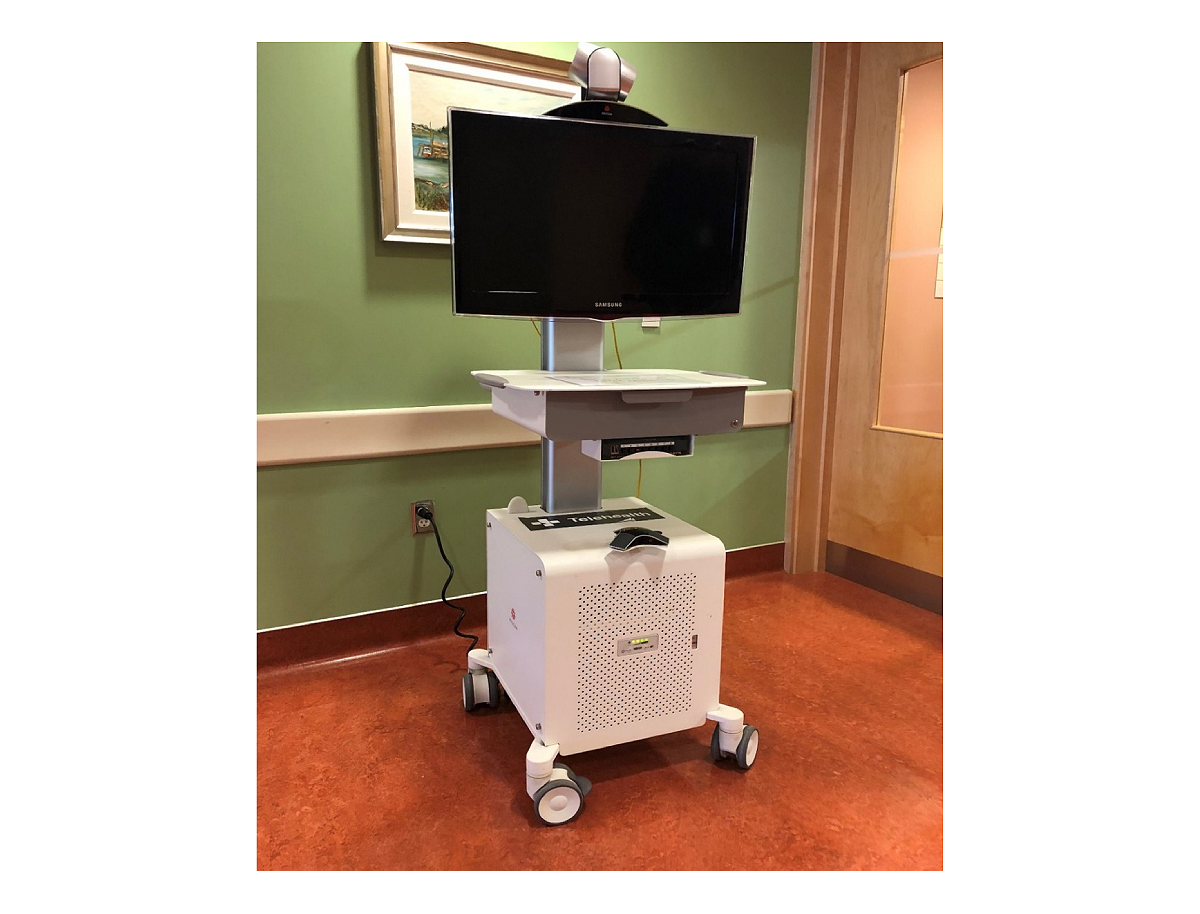
Mobile telemedicine cart capable of real-time audiovisual transmission.

The use of telemedicine to provide outpatient clinical care is common and usually involves a single patient visit and a few health care providers. The imbalance in use of pediatric beds in Alberta warrants testing innovative solutions to provide equitable and patient- and family-centered care to pediatric patients in nonurban and rural parts of Alberta. Therefore, *Telemedicine Rounding and Consultation for Kids* (TRaC-K), a telemedicine-facilitated model for inpatient pediatric clinical care, is in development and will be piloted between ACH and MHRH. This model was jointly developed by the team of health care providers from ACH and MHRH, as well as administrators and technical experts at AHS. TRaC-K will enable health care providers at ACH to partner with their counterparts at MHRH to provide inpatient clinical care for pediatric patients from the MHRH catchment area. The model promotes regional site access to tertiary care providers and enhances collaboration and communication between care teams. The potential patients for the TRaC-K model will be those who are either admitted at MHRH and could benefit from TRaC-K to receive care at MHRH or those from the MHRH catchment area who are admitted at ACH but are stable and could benefit from TRaC-K for potential early transfer back to MHRH to complete their remaining treatment. These patients will be identified by health care providers from both sites. A telemedicine cart with capability to provide real-time audiovisual transmission will be used for TRaC-K (Figure 1). Textbox 1 lists the key features of the TRaC-K model.

**Key features of Telemedicine Rounding and Consultation for Kids (TRaC-K).**

**TRaC-K: key features**

- Daily inpatient census and patient rounds
  Census: Monday to Friday; health care teams review patients eligible for TRaC-K at both sites
  Patient-focused consultation or rounds regarding the care of a patient at 1 site involving teams at both sites and may occur at the bedside with the patient and family included in care planning when able and appropriate
- Telemedicine-facilitated
  Real-time audiovisual transmission using telemedicine cart (Figure 1)
- Tertiary–regional collaboration
  Collaboration between Alberta Children’s Hospital (tertiary) and Medicine Hat Regional Hospital (regional)
- Multidisciplinary teams
  General pediatricians, subspecialists, allied health professionals, nurses, and nurse educators (based on patient needs)

The patient-centered–care approach of the TRaC-K model will benefit patients from regional catchment areas who will be able to access tertiary hospital–level care expertise and resources within their own region without having to travel to the tertiary-level hospital. Families will also benefit by saving travel time, minimizing time away from work, and being able to stay better connected to their community support networks.

Inpatient care at both tertiary and regional sites is provided within a complex health system with many providers and occurs independently with limited communications through telephone for specialist physician consultation related to the care of patients. Therefore, it is important to apply rigorous methods to understand the potential influences on behavior in the context in which it will occur and understand the mechanisms of change even before implementation of the intervention. There exist several models and frameworks for assessing and identifying barriers and enablers to implementation of eHealth interventions, but their focus lies on technological aspects rather than behavioral determinants at the individual level. In the TRaC-K model, technology acts as the facilitator for the clinical model through behavior change. Implementing the TRaC-K model requires change in individual and collective behaviors of all stakeholders involved (ie, physicians, nurses, allied health providers, hospital and unit managers and administrators, patients, and families). Therefore, to systematically identify the stakeholders’ perceived barriers and enablers to the implementation of the proposed TRaC-K model of inpatient pediatric care, we used the Theoretical Domains Framework (TDF). The TDF provides a theoretical lens to identify barriers and enablers to change to inform implementation of an intervention in local contexts. The TDF consists of 84 theoretical constructs that are refined into 14 domains [13,14]. The TDF has been used widely to identify behavioral determinants to implementation of health care interventions [15-17].

Therefore, a study identifying perceived barriers and enablers such as health care providers’ skills, their perceptions of compatibility of the TRaC-K model, and their motivations to provide virtual care would help in the implementation of TRaC-K, especially in the local context of Canada’s first and largest province-wide, fully integrated health system. A paucity of evidence in the scientific literature regarding the implementation of such interventions undermines telemedicine’s potential to provide high-quality, cost-effective, patient- and family-centered, and equitable clinical care [18,19]. To our knowledge, no other study has comprehensively studied barriers and enablers to such telemedicine models of inpatient pediatric clinical care.

The COVID-19 pandemic has exponentially increased the uptake of telemedicine in clinical care; however, to our knowledge, TRaC-K is the first telemedicine-facilitated model to provide inpatient clinical care for pediatric patients. Therefore, the objective of this study is to conduct a systematic and theory-informed identification of barriers and enablers to the implementation of the TRaC-K model between ACH and MHRH.

## Methods

### Design

This study is part of a larger multiphase project to develop, implement, and evaluate a virtual tertiary–regional telemedicine rounding and consultation model of inpatient pediatric care. This is a qualitative descriptive study guided by the TDF. The protocol of this study has been published elsewhere [20].

### Ethics

Ethical approval for this study was obtained from the Conjoint Health Research Ethics Board at the University of Calgary (REB17-1435). Administrative approval for this project was also obtained from AHS.

### Setting

Participants were selected from ACH and MHRH. ACH is located in Calgary; with 141 beds, it is the largest tertiary pediatric hospital in Alberta. MHRH is located in the city of Medicine Hat, Alberta. It is a 325-bed regional hospital with 10 dedicated beds for pediatric patients, excluding the neonatal intensive care unit.

### Participants

We recruited a stratified purposive sample of clinical stakeholders at ACH and MHRH. In addition, we invited family caregivers of pediatric patients with a history of availing inpatient medical services at both ACH and MHRH in the last 3 years to participate in this study. At ACH, we recruited participants by sending an email describing the study, which included an invitation to participate, to unit and allied health managers as well as physician leaders to distribute among their respective teams. We also contacted administrators, including senior leaders at ACH, through email. At MHRH, we recruited participants by sending an email describing the study, which included an invitation to participate, to site pediatric managers and the physician coinvestigator of the study who also distributed it among their respective teams. At ACH, pediatricians and Family Advisory Council members suggested the names of family caregivers meeting our criteria as potential study participants; at MHRH, a single pediatrician obtained the consent to contact from family caregivers to participate in this study. We contacted these family caregivers through email. Interested participants replied to the emails, and we contacted them to set up an in-person interview or telephone interview or focus group. Participant recruitment continued until saturation. We offered a CAD $15 (US $12) gift card honorarium to all participants for their participation in the study.

### Interview Topic Guide

The TDF informed the interview guide, with 2-4 questions formulated to explore each of the 14 TDF domains (Multimedia Appendix 1). Investigators with expertise in implementation science (CC and JC) provided guidance to develop the interview guide. Next, members of the research team reviewed the guide to refine it further. We used the same guide for the focus groups, but fewer key questions were asked to initiate the discussion on different TDF domains. We modified some of the questions for the interviews with family caregivers of pediatric patients.

### Procedure

A single interviewer (SB), who has expertise and experience in qualitative research, conducted all the interviews and focus groups at ACH and MHRH. For the focus groups, a notetaker accompanied the interviewer to take field notes. Before each in-person interview or focus group, we reviewed the written consent form, and each participant signed it. We excluded administrators from both the focus groups to prevent the power differential from influencing group dynamics during the focus group discussions. For the telephone interviews, we emailed the consent form to the participants before the interview and obtained written consent at the beginning of the interview. In addition, all participants from ACH and MHRH, including family caregivers, completed a brief demographic form. Individual interviews lasted 30-60 minutes, and each focus group lasted approximately 50 minutes. We audio recorded and transcribed verbatim all the interviews and focus group discussions. Subsequently, we imported all the transcripts into NVivo software (version 11; QSR International) to code, organize, and manage the data to facilitate analysis.

### Data Analysis

Before analyzing all the data, 2 reviewers from the research team (SB and CC) independently coded 2 randomly selected transcripts to discuss consistency in coding and develop a codebook. Discrepancies were resolved through discussion. On the basis of this discussion, a codebook was developed, and a single reviewer (SB) coded the remaining transcripts using this codebook.

We analyzed data in 3 steps. First, a directed content analysis approach [21] was used to categorize similar belief statements into each of the 14 TDF domains. We cross-indexed similar statements in multiple domains if they were relevant to more than one domain. Second, we used an inductive coding approach [22] to group similar belief statements to form subthemes within the initial coding scheme of the 14 TDF domains. Finally, as a study team, we further examined these subthemes to aggregate and reword before categorizing them into barriers and enablers. To prepare the results, we tabulated the subthemes within each TDF domain and tabulated larger themes into barriers and enablers separately. Quotations illustrating core beliefs are used to highlight subthemes in each domain.

## Results

### Interviews and Focus Groups

All the interviews and focus groups were held over a 10-month period (from November 2017 to August 2018). We conducted 31 interviews and 2 focus groups. At ACH, we conducted 15 semistructured individual interviews and 1 focus group. At MHRH, we conducted 16 semistructured individual interviews and 1 focus group. A total of 29 interviews were held face to face, whereas 2 interviews were conducted by telephone because of the inability of the participants to travel to either ACH or MHRH. The ACH focus group consisted of 5 participants, whereas the MHRH focus group included 6 participants. Both focus groups included pediatricians, nurses, and other allied health professionals. Thematic saturation where no new information was achieved was reached after interviewing 42 participants (see Table 1 for characteristics of the participants).

**Table 1:** Characteristics of study participants (N=42).

Characteristics and category		Participants
**Gender, n (%)**		
	Male	3 (7)
	Female	39 (93)
**Site, n (%)**		
	Alberta Children’s Hospital	20 (48)
	Medicine Hat Regional Hospital	17 (40)
	Family members	5 (12)
**Position, n (%)**		
	Administrator	7 (16)
	General pediatrician	8 (19)
	Pediatric subspecialist	2 (5)
	Nurse	10 (24)
	Allied health professional	10 (24)
	Family member	5 (12)
**Focus group participants, n (%)**		
	Alberta Children’s Hospital	5 (45)
	Medicine Hat Regional Hospital	6 (55)

During data analysis, key statements demonstrating the beliefs of participants were attributed to each TDF domain. Next, the statements in each domain were grouped to form subthemes. Multimedia Appendix 2 lists all 14 domains of the TDF, their definition, subthemes, and representative quotes for each subtheme.

### Barriers and Enablers to Implementing TRaC-K

The subthemes identified in each domain (Multimedia Appendix 2) were further categorized and tabulated into barriers and enablers. This study identified 4 barriers and 4 enablers to the implementation of the TRaC-K model, which are presented in Table 2.

**Table 2:** Telemedicine Rounding and Consultation for Kids (TRaC-K) barriers and enablers.

Themes and subthemes				Theoretical Domains Framework domain source
**Barriers**				
	**Awareness of telemedicine**			
		Limited awareness about the use of telemedicine in pediatric clinical care	1	
	**Skills to provide virtual clinical care**			
		Lack of skills to communicate over the screen	2	
		Lack of clinical assessment skills to provide care over the screen	2	
		Lack of technical skills	2	
	**Processes and resources to support TRaC-K**			
		Unclear processes as a potential source of harm	5, 6	
		Considering challenging clinical circumstances	4, 6, 10, 11	
		Physical environment	10	
		Absence of dedicated personnel	14	
		Difficulties in scheduling	11	
		Paucity of professional guidelines	3	
	**Provider roles and responsibilities**			
		Concerns about clear roles and responsibilities	4	
		Lack of workflow integration	4, 6	
		Increased workload and competing priorities	11, 7	
**Enablers**				
	**Motivation to provide care closer to home**			
		Desire to provide care closer to home	3	
		Confidence in TRaC-K	5	
		High importance	8	
		Excitement	13	
	**System resource stewardship**			
		Balancing provincial resources	3, 6	
		Ability to provide tertiary-level care at regional sites	4	
		Redistribution of patient load and resources; care closer to homes	3, 7	
	**Site and practice compatibility**			
		Compatible with current practice	9	
		Buy-in from key stakeholders	12	
		Education for potential TRaC-K users	14	
	**Motivation to strengthen tertiary–regional relationships**			
		Opportunity for trust building	3, 6	
		Opportunity for educational exchange	3, 7	

### Barriers

#### Theme 1: Awareness of Telemedicine

Overall, there was limited awareness about the use of telemedicine in pediatric clinical care. All participants were aware of telemedicine in some form such as telephone calls or Skype video meetings among health care providers or between health care providers and patients. Most respondents had heard about telemedicine in adult care or in other jurisdictions of Canada or in the United States. Participants from MHRH were aware of outreach clinics where Medicine Hat patients have direct telemedicine consults with their pediatric specialists at ACH, but MHRH pediatric unit staff are not involved in these consults. Some of the participants reported that they have used or heard of telemedicine being used to provide specific types of care, such as transition of care or use of synchronous audiovisual telemedicine by the patient transport team.

At the time of the interviews, awareness about using synchronous audiovisual technology for rounding and consultation in inpatient pediatric care was very low. Almost all participants reported that this was the first time they were hearing about a telemedicine-based model of inpatient pediatric care.

#### Theme 2: Skills to Provide Virtual Clinical Care

Participants identified a number of skills that were currently lacking and would need to be developed to use TRaC-K successfully. All respondents acknowledged the difference between in-person and virtual communication; therefore, they underlined effective communication as an important skill for the TRaC-K model of care. Family members also highlighted that lack of proper communication skills would hamper developing trust and building rapport between patients and health care providers and among health care provider teams. Health care providers at both sites reported that well-organized communication with an ability to verbalize findings, being concise with the information, and framing proper questions would be essential components of communication skills required to provide care through the TRaC-K model. MHRH providers also pointed out that as health care providers at a regional site, MHRH team members would sometimes have to be assertive during the TRaC-K rounds.

In terms of using clinical skills in a virtual health environment, health care providers at ACH believed that they would have to rely on team members at MHRH for clinical observations. In addition, they would have to improve their skills to virtually assess patients over the screen. In contrast, health care providers at MHRH believed that because of TRaC-K, they would have to handle patients with higher acuity; therefore, they might have to advance their assessment skills to make decisions about a transfer to ACH. They might also have to advance their practical skills to perform new or less familiar procedures.

Finally, participants also stressed the importance of technical skills to handle the telemedicine cart and their ability to troubleshoot any technical issues during rounds.

#### Theme 3: Processes and Resources to Support TRaC-K

Participants identified that lack of clear processes and resources could be an important barrier to successfully use TRaC-K. Most of the participants did not think there was any harm in providing care using the TRaC-K model, but several participants identified some potential harms such as overreliance on technology and incorrect use of this mode of providing care if processes were unclear. Health care providers mentioned a need for specific processes for using TRaC-K in challenging clinical situations as well as ensuring resources to support care in these situations.

Participants highlighted the inability to perform physical examinations and potential miscommunication as sources of adverse outcomes for patients. Health care providers raised concerns about potential delay or failure in recognizing and managing patients whose condition was deteriorating because patients’ conditions may change quickly. Health care providers mentioned that because of lack of resources at MHRH, their decision to directly transfer patients to ACH instead of using TRaC-K would be influenced by the need to treat patients classified as acute, especially those with life-threatening emergencies such as respiratory arrest and cardiac arrest as well as those requiring intubation. In addition, health care providers listed clinical situations when it would be difficult to provide care using TRaC-K, such as those requiring *hands-on* care by specialists as well as those involving mental health issues, palliative care, child abuse cases, patient counseling, and patients with complex care needs. Therefore, it was suggested to establish good criteria to determine the kinds of patients who are appropriate to receive care using TRaC-K and those who are not appropriate.

Health care providers also noted several family factors that could contribute to challenging situations, such as non–English-speaking patients, cultural minorities, and families with significant stressors. They highlighted the importance of acceptance of TRaC-K by patients and their families. Participants suggested that one of the ways to mitigate these issues was to educate patients and their families about the TRaC-K model by explaining the reasons for using TRaC-K when they are admitted at MHRH or ACH and addressing any concerns they might have. Other forms of support, such as the Language Line telephone interpreting service to provide care for non–English-speaking patients and billing codes for MHRH pediatricians, were also identified by a few MHRH health care providers.

Specific resources such as the setup of the physical environment and availability of personnel for coordination of TRaC-K were mentioned by many participants. According to participants, factors related to the physical environment, such as the ease of moving the TRaC-K cart among different rooms, size of the patient rooms, and privacy in these rooms, might hinder the use of TRaC-K.

According to many participants, the implementation of TRaC-K involves several logistical issues such as testing technology, scheduling, and coordinating between the 2 sites. Scheduling TRaC-K rounds as part of the workflow and background coordination were consistently highlighted as some of the major challenges. Participants stressed the importance of having support in place before providing care using the TRaC-K model, and they indicated that having dedicated TRaC-K coordinators at ACH and MHRH would address many barriers.

All participants were asked if they were aware of any practice guidelines from their professional organizations regarding telemedicine-facilitated care. Interestingly, almost all were unsure or unaware of any relevant practice guidelines. Many of them guessed that the use of telemedicine would be encouraged by their professional organization but were not able to provide any concrete information about what type of guidelines their organizations want them to adhere to while providing telemedicine-facilitated inpatient care.

#### Theme 4: Provider Roles and Responsibilities

Health care providers at both ACH and MHRH stressed the importance of having clear roles and responsibilities. Considering the potential for conflict and medicolegal issues, health care providers wanted clear guidelines on who holds the primary responsibility for the patient, especially if there is disagreement among health care providers at ACH and MHRH in the presence of families.

Health care providers from ACH also raised the issue of their lack of understanding of the capabilities of regional hospitals and the staff at MHRH. The need to integrate TRaC-K workflow into current clinical structures was also highlighted by participants. Health care providers at MHRH mentioned that pediatricians there provide care at their private clinics as well as at the inpatient unit and emergency department at MHRH; therefore, finding time to schedule their TRaC-K rounds would be challenging. Similarly, health care providers at ACH and MHRH also emphasized the importance of scheduling TRaC-K rounds in advance. Health care providers at ACH also did not want to be torn between multiple sites because this may have a negative impact on the care they provide for their own patients at ACH.

Participants were asked to imagine the potential changes that would occur with the implementation of TRaC-K. Health care providers at MHRH expressed that TRaC-K would increase their workload because they would see more patients classified as acute, and because of TRaC-K, MHRH patients transferred to ACH might be transferred back to MHRH, resulting in higher use of the pediatric unit at MHRH and increasing their workload. Participants also shared their thoughts on the situation that would arise if TRaC-K was not implemented. Without TRaC-K, the status quo would continue, resulting in continuation of current practices, including calling ACH by telephone for specialist physician consultation, patients and families traveling to ACH, and imbalance in bed occupancy.

Other competing priorities were also as seen as a potential barrier. Health care providers at MHRH mentioned that they have fewer pediatricians than ACH, and most of them have their own private clinics; therefore, they have to provide care at different locations, and accordingly sometimes other competing priorities might act as a barrier to using TRaC-K.

Additional issues such as the comfort level of health care providers at MHRH to handle patients with complex care needs and non–English-speaking patients were also mentioned as some of the circumstances under which it would be difficult to use TRaC-K. Some patient-related challenges in the use of TRaC-K were also pointed out. According to a health care provider at ACH, some patients “like” to be at ACH for various nonclinical reasons such as an opportunity to shop and visit the city.

### Enablers

#### Theme 1: Motivation to Provide Care Closer to Home

Participants at ACH and MHRH stressed the importance of providing care closer to home, especially for pediatric patients. Participants believed that pediatric patients and their parents develop their social support mechanism in their own communities; therefore, taking patients to ACH disconnects them from such social support. The long commute (3-4 hours, one way) between MHRH and ACH and the dangers associated with such long-distance travel, especially during snowy winter months, was stated as another reason for providing care closer to home.

Most of the participants showed high confidence in TRaC-K and affirmed that it would not drastically change the current practice of providing care but rather enhance it. During the focus group at ACH, a few health care providers cautioned against overvaluing TRaC-K as a total replacement for face-to-face care. Despite several potential sources of harms having been identified, participants supported TRaC-K and expressed that the benefits of using TRaC-K would outweigh potential harms or negative consequences.

Participants also felt that the TRaC-K project was of high importance. They were asked to rate the importance of providing care through the TRaC-K model on a scale of 1-10, with 10 being very important. The score given by participants ranged from 5 to 10; most rated it as 8 or 9. The reasons for giving high scores were the TRaC-K model’s ability to keep patients and families within local communities and its potential to improve the quality of care. Some of the reasons cited for lower scores were a potential increase in workload and skepticism about the TRaC-K model’s usefulness.

In general, participants were excited about the TRaC-K model mainly because they want to try something new and TRaC-K offers them a novel way to care for their patients. However, without having tried it, some participants mentioned being optimistically cautious.

#### Theme 2: System Resource Stewardship

Participants acknowledged the importance of telemedicine-facilitated pediatric care in promoting sustainability from a health system’s perspective. Participants were aware of overcapacity at ACH and underuse of pediatric beds at MHRH; therefore, they recognized that the TRaC-K model would help in balancing the patient load. According to participants, TRaC-K would also facilitate transition of care for patients with complex chronic conditions from ACH to MHRH. Therefore, TRaC-K was viewed as a mechanism to address this imbalance and to advocate for additional resources for regional sites such as MHRH. None of the participants mentioned any personal monetary incentives to use the TRaC-K model of inpatient clinical care. However, the potential cost saving for AHS was mentioned as a financial incentive at the system level.

Providing care at regional sites when possible was deemed important for the system and for families. All participants reiterated the important role of TRaC-K in providing patient- and family-centered care in local communities for patients from regional sites. Other key benefits such as prompt expert care, faster diagnosis and treatment, and better quality of care were also mentioned. Health care providers acknowledged that using TRaC-K would help to provide family-centered care, and if patients and families at MHRH felt that they were not at the best hospital, then joint rounds using TRaC-K in their presence would show that ACH and MHRH were working as 1 team to provide the best care for their child. Family members mentioned the potential reduction in wait times to see a specialist as an incentive for them to use the TRaC-K model.

Participants described various incentives for them to use TRaC-K from their own perspectives. The biggest incentive for health care providers was the professional satisfaction gained by providing best-quality care closer to the patient’s home.

#### Theme 3: Site and Practice Compatibility

Participants overwhelmingly agreed that TRaC-K would be compatible with their practice because it aligns with their motivation to provide patient- and family-centered care and technology is slowly changing the way care is provided; therefore, with a few modifications, they could easily incorporate TRaC-K into their practice or adapt their practice to use TRaC-K.

Participants highlighted that buy-in from all health care providers, especially from key stakeholders such as physicians and administrators, would be essential for successful implementation of TRaC-K. A participant even considered the interviews and focus groups for this study as an educational opportunity for them to know more about TRaC-K.

Many participants alluded to the importance of educating potential users of TRaC-K, including health care providers at ACH and MHRH and patients and their families. The educational activities mentioned were creating educational material, performing hands-on trials with technology, and practicing in mock scenarios. It was suggested that to promote family-centered care, families should be engaged right from the beginning, with explanations of the TRaC-K model provided to them verbally or by using pamphlets and of what they should expect during their stay at MHRH, instead of a screen being wheeled directly into their room and placed in front of them.

#### Theme 4: Motivation to Strengthen Tertiary–Regional Relationships

Participants from MHRH affirmed that having access to, and frequent interactions with, the teams from ACH would help build trust between regional and tertiary health care providers. Participants at MHRH pointed out that having access to tertiary health care providers, including specialists at ACH, through TRaC-K would enhance patients’ and families’ trust in MHRH and encourage them to seek care at MHRH instead of traveling to ACH, especially in situations where visiting ACH was unnecessary.

During joint rounds, clinical teams from ACH would be able to disseminate knowledge about new treatment guidelines, procedures, and protocols for handling patients with complex care needs among their peers at MHRH. ACH providers would also be able to learn about the challenges and capacities of regional sites. Participants from MHRH also viewed TRaC-K as an opportunity to learn and receive more support from their colleagues at ACH. Therefore, implementation of the TRaC-K model was considered a great learning opportunity for health care providers at both ACH and MHRH, which would enable both sides to share clinical knowledge. Regular communication between regional and tertiary health care providers would also serve as additional support for newly trained clinicians moving to regional sites because they would be able to receive second opinions and have a new set of eyes looking at their patients.

## Discussion

### Principal Findings

Our study identified 31 major subthemes in 14 domains, ranging from administrative issues to specific clinical conditions. These subthemes were further aggregated into major themes and categorized into 4 barriers and 4 enablers. Our findings suggest that most of the barriers concern uncertainties associated with potential hands-on, day-to-day use of TRaC-K. We anticipate that some of these barriers will be mitigated once the TRaC-K model pilot begins. On the basis of these barriers, dedicated TRaC-K coordinators will be hired to address administrative issues such as scheduling, providing technical support, and streamlining the process to reduce some of the workload. Some of the barriers identified in this study, such as lack of information technology skills and paucity of professional guidelines, are consistent with those identified in a study from Australia [12]. The ongoing COVID-19 pandemic has significantly affected the delivery of health care. Travel restrictions and the burden on tertiary sites have created a unique opportunity to use telemedicine. The urgency of providing efficient clinical care during this pandemic has mitigated many barriers to using telemedicine; however, the use of telemedicine in the postpandemic era remains uncertain. Many health care systems have realized the importance of telemedicine but still struggle to use it as a long-term solution [23]. Thus, the findings of this study remain timely and relevant.

On a positive note, the enablers demonstrate the willingness of health care providers to embrace the change to provide patient- and family-centered care. The enablers were also associated with participants’ desire to change the current imbalance in health care use between ACH and MHRH and a positive attitude toward TRaC-K’s potential to change the status quo and probably open new opportunities for educational exchange and trust building between tertiary and regional sites. ACH and MHRH are part of AHS, a single province-wide health authority, which might have helped participants to understand the importance of rebalancing resources between tertiary and regional sites. The potential of these enablers will be harnessed to encourage health care providers to use TRaC-K.

Next, the findings from this study will be used to map potential barriers and enablers to the behavior change techniques (BCTs) from BCT Taxonomy version 1, which is a standardized list of 93 hierarchically clustered BCTs [24]. The BCT-mapping exercise will be conducted with the key stakeholders. The BCTs that are likely to change behavior will be used as ways to mitigate barriers and harness the potential of enablers. Finally, the revised TRaC-K model will be piloted between ACH and MHRH for 1 year. The results of the TRaC-K pilot study will determine its feasibility and scale-up throughout the province.

The province-wide application of the TRaC-K model may increase regional pediatric bed use at multiple regional sites, thereby having a positive impact on the current tertiary overcapacity crisis in Alberta. In addition to addressing the imbalance in the use of pediatric capacities between regional and tertiary hospitals, it is anticipated that this project will enhance tertiary–regional collaboration, thereby supporting numerous other provincial goals such as provincial pediatric guideline implementation and pediatric workforce sustainability. AHS has created various strategic clinical networks (SCNs) as engines for research and innovation as well as to act as vehicles for translating evidence into practice to improve patient care and health system performance. This project is supported by the Maternal, Newborn, Child & Youth SCN. Therefore, the findings of this study will be disseminated as pediatric health services research–generated knowledge through the Maternal, Newborn, Child & Youth SCN to drive the pilot and scale-up of TRaC-K in Alberta.

Interventions such as the TRaC-K model could fail to achieve the expected outcomes because of the lack of a scientific approach to identify and address factors such as barriers and enablers before implementation. This study contributes a comprehensive and systematic inquiry into perceived barriers and enablers to implementing telemedicine to the body of scientific literature. The evidence generated from this study would not only benefit other health care systems interested in implementing telemedicine-facilitated interventions to provide inpatient clinical care, but also serve as a publication to guide other research teams undertaking similar research to identify barriers and enablers for similar interventions within their own contexts.

The systematic and theoretical framework–driven approach to identify potential barriers and enablers to the TRaC-K mode is a clear strength of this study. In addition, a major strength of this study was the diversity of perspectives captured through interviews and focus groups. The inclusion of families of pediatric patients who frequently travel between ACH and other regional sites provided patient- and family-centric insights regarding the TRaC-K model. However, the findings of this study must be interpreted keeping some limitations in mind. First, this study was conducted before the COVID-19 pandemic; therefore, some of the barriers and enablers might have changed. Methodologically, the TDF is a framework; hence, it only describes empirical phenomena by classifying them into sets of domains. The TDF neither describes nor provides an explanation for a phenomenon; nor does it specify a relationship among its domains. Although we excluded administrators from the focus groups to mitigate the potential power imbalance, pre-existing professional relationships might have influenced the opinions of some of the participants of these focus groups. Finally, recruitment through stratified sampling required contacting family caregivers meeting the eligibility criteria through their pediatricians, which might have resulted in selection bias.

### Conclusions

This systematic inquiry into perceived barriers and enablers to the implementation of TRaC-K helped us to gain insights from health care providers’ and family members’ perspectives. We are optimistic that the implementation of TRaC-K will be successful based on the enablers identified through this study. Enablers such as motivation to provide care closer to pediatric patients’ homes and to adjust the imbalance in health care resource use will play a key role in the implementation of TRaC-K. The association of barriers with the uncertainties concerning day-to-day use of TRaC-K will enable us to address these barriers by creating clear processes and providing support through dedicated staff. Finally, these findings will inform the development and implementation of telemedicine-based interventions in other parts of Canada and beyond.

## Appendix

Multimedia Appendix 1 Interview guide for health care providers.

Multimedia Appendix 2 Subthemes identified in each domain of the Theoretical Domains Framework.

## Abbreviations

ACH: Alberta Children’s Hospital
AHS: Alberta Health Services
BCT: behavior change technique
ICT: information and communication technology
MHRH: Medicine Hat Regional Hospital
MNCY SCN: Maternal, Newborn, Child & Youth Strategic Clinical Network
SCN: strategic clinical network
TDF: Theoretical Domains Framework
TRaC-K: Telemedicine Rounding and Consultation for Kids
