# Supplementary material for: Using the Theoretical Domains Framework to Identify Barriers and Enablers to Implementing a Virtual Tertiary–Regional Telemedicine Rounding and Consultation for Kids (TRaC-K) Model: Qualitative Study
Source: J Med Internet Res. 2021 Dec 22;23(12):e28610. doi: 10.2196/28610 (PMC8734914; doi:10.2196/28610)
Supplement: Multimedia Appendix 2 [file jmir_v23i12e28610_app2.docx]

**Multimedia Appendix 2.** Sub-themes identified in each domain of the Theoretical Domains Framework.

| Domain (definition) and subthemes | | Quotes |
| --- | --- | --- |
| 1. Knowledge (an awareness of the existence of something) | | |
|  | Awareness about telemedicine | “For example, we’ve done some transfer meetings through telemedicine um but not really with a patient present and not from a diagnostic perspective, more from just information sharing is my, the capacity that I’ve used it into date.” [HCP03, subspecialist, ACH^a^] |
|  | Lack of awareness about telemedicine in pediatrics | “I know that we do have some patients who come and see some of our specialists in Calgary in our telemedicine room...yeah. I don’t have an active role in those appointments...but I see them go on.” [HCP29, nurse, ACH] |
| 2. Skills (an ability or proficiency acquired through practice) | | |
|  | Communication skills | “Talking to a monitor is going to be different than face-to-face. You’ll have to be organized with what we need to be presenting to our doctors there as we probably have limited time with them. So, I guess organizational communication skills to make sure that we’re communicating the important facts...and make sure that we’re not missing out on anything.” [HCP29, nurse, ACH] |
|  | Clinical skills | “...the ability to listen and to make observations through the tele-video and audio. There’s got to be that ability and perhaps even some training for people to know how to talk, listen, provide advice but even assess patients over video and audio.” [HCP08, administrator, ACH] |
|  | Technical skills | “I could review on how to actually use the technology. Yeah sure, like moving the camera, actually setting it up. I feel like things always go wrong and we just call IT.” [HCP06, allied health professional, ACH] |
| 3. Social and professional role and identity (a coherent set of behaviors and displayed personal qualities of an individual in a social or work setting) | | |
|  | Providing care closer to home | “It would be nice for the family and uh...patient to be closer to home to their support systems and um...and to their life, so if a parent needs to attend to other children um...or continue to work or whatnot, they can still do that....” [HCP13, pediatrician, MHRH^b^] |
|  | Providing care closer to home | “The travel is...is difficult for parents especially in the wintertime.” [Family02] |
|  | Balancing provincial resources | “...as far as the health care systems goes, this is an incredible uh...money-saving opportunity.” [HCP05, pediatrician, ACH] |
|  | Lack of guidelines from professional organizations | “Oh, I have no idea. I [pause] I don’t know. I would think that they [professional organization] would um support it [telemedicine].” [HCP04, nurse, ACH] |
|  | Learning and trust building | “...also trust perspective with the family, if they know that they’re connecting with us, they see it or know that that’s happening, I think that enhances again their willingness and their ability to stay local and not feel like they’re missing some important diagnostic piece or something you know magic that happens at a tertiary care center.” [HCP16, pediatrician, ACH] |
| 4. Beliefs about capabilities (acceptance of the truth, reality, or validity about an ability, talent, or facility that a person can put to constructive use) | | |
|  | Clear roles and responsibilities | “We need to be really clear again is what is my role versus what is the attending physician’s role at the site and who holds the responsibility is the biggest issue.” [HCP17, pediatrician, ACH] |
|  | Workflow integration | “I think that organizational perspective in the same way and you might have a designated person that’s sort of the telehealth person of the day or something like that that would be responsible for organizing whatever is needed for, for those rounds.” [HCP05, pediatrician, MHRH] |
|  | Tertiary-level care at regional sites | “Having them involved with the rounds will help show that it’s one team, one care, right, for their child so....” [HCP22, administrator, MHRH] |
| 5. Optimism (the confidence that things will happen for the best or that desired goals will be attained) | | |
|  | Cautiously confident | “I’d rather not overvalue what it is because I’m worried that it doesn’t replace being able to actually stand in front of somebody and look at them and assess them. Like I don’t think that it’s still equal level of care and I don’t think that it’s fair to the families to think it is.” [HCP23, nurse, ACH] |
| 6. Beliefs about consequences (acceptance of the truth, reality, or validity about outcomes of a behavior in a given situation) | | |
|  | Redistribution of patient load | “We have a huge discrepancy in occupancy rates between the rural centers and uh our center and so creating that balance I think is the right thing to do.” [HCP01, administrator, ACH] |
|  | Supporting regional provider | “The second thing that is very good about it is, is the ability for the education...um...in that, in that there would be an exchange of experience, uh standards, ideals between the two sites that would escalate.” [HCP10, subspecialist, ACH] |
|  | Supporting regional provider | “Maybe our graduates would feel more comfortable and confident going to regional centers, so that would be really interesting if it’s part of the solution to that staffing.” [HCP07, pediatrician, ACH] |
|  | Potential sources of harm | “Just lost in translation or, you know, you maybe have it written down here, but you don’t quite communicate it properly.” [HCP21, pediatrician, MHRH] |
|  | Potential sources of harm | “My biggest worry is what impact does that have on the patient, are we delaying [pause] care that we might not know at the time but suddenly in an hour crap, we’ve delayed them now.” [HCP39, nurse, MHRH] |
| 7. Reinforcement (increasing the probability of a response by arranging a dependent relationship or contingency between the response and a given stimulus) | | |
|  | Professional satisfaction | “Other than the fact that we wanna provide the best care to our patients so that would, that would be the incentive but there's no, I don’t think there's any other incentive um administratively or, or anything else.”[HCP16, nurse, MHRH] |
|  | Increase in workload | “Workload is maybe a concern. Yeah, because we are only a 10-bed unit and we run six to seven already so sometimes when we’re transferring out it’s because we don’t always have the staff available....” [HCP29, nurse, MHRH] |
| 8. Intentions (a conscious decision to perform a behavior or a resolve to act in a certain way) | | |
|  | High importance | “I think that the reason a nine is we haven’t worked out the process pieces of this yet so I think once I, once we work on the process pieces, that would help more.” [HCP31, allied health professional, MHRH] |
| 9. Goals (mental representations of outcomes or end states that an individual wants to achieve) | | |
|  | Compatibility | “I think it would be compatible. I think you could make it work. It would be a change and it would be something that we would have to adapt to.” [HCP29, nurse, ACH] |
| 10. Memory, attention, and decision processes (the ability to retain information, focus selectively on aspects of the environment, and choose among 2 or more alternatives) | | |
|  | Specific clinical situations | “I suppose certain, certain subspecialties such as cardiology would be difficult because a lot of what they might want us to happen or to do for the patient such as an echo we still wouldn’t be able to do.” [HCP34, pediatrician, MHRH] |
|  | Specific clinical situations | “If there’s a lot of counseling that needed to be done that would be quite...could be quite difficult.” [HCP11, allied health professional, ACH] |
|  | Specific clinical situations | “I think when a patient is decompensating.” [HCP17, nurse, MHRH] |
|  | Patient- and family-related issues | “Emergency type situations would not work...I don’t think.” [Family01] |
|  | Patient- and family-related issues | “Obviously, number one is the family doesn’t agree to participate in that model of care because I’m not sure it’s something we can force a family to do.” [HCP07, pediatrician, ACH] |
|  | Physical environment | “Can I move that [TRaC-K^c^ cart] about to, to different rooms, is it easy to move that, is there enough room in the, in the rooms to have this, the equipment.” [HCP05, pediatrician, ACH] |
| 11. Environmental context and resources (any circumstance of a person’s situation or environment that discourages or encourages the development of skills and abilities, independence, social competence, and adaptive behavior) | | |
|  | Scheduling | “Definitely time, routines might have to change.*”* [HCP24, nurse, MHRH] |
|  | Patient acuity | “Any cardiac arrest I feel like those would all need to be sent because those, we don’t have capabilities of like a PICU [pediatric intensive care unit].” [HCP29, allied health professional, MHRH] |
|  | Other competing priorities | “It would be a very uncomfortable position to be in to know that I have a high priority patient I need to go see and trying to decide between coming to the scheduled telemedicine session versus seeing that high priority patient.” [HCP07, pediatrician, ACH] |
| 12. Social influences (those interpersonal processes that can cause individuals to change their thoughts, feelings, or behaviors) | | |
|  | Positive attitude | “Oh, Dr [name]’s all for it, she loves it, yeah, she loves it, she definitely is a strong believer in this and it’s for the same reasons that I am I would say.” [HCP19, nurse, MHRH] |
|  | Uptake from key stakeholders | “I would think that unless you have buy-in from everyone, um then that's gonna be a bit of a, a bit of a, an issue, right...I think that the buy-in from physicians is huge.” [HCP16, pediatrician, ACH] |
| 13. Emotion (a complex reaction pattern, involving experiential, behavioral, and physiological elements, by which the individual attempts to deal with a personally significant matter or event) | | |
|  | Excitement | “A little bit of nervousness just because it’s [a] change but also excited at the possibilities.” [HCP07, pediatrician, ACH] |
| 14. Behavioral regulation (anything aimed at managing or changing objectively observed or measured actions) | | |
|  | Dedicated person | “I think that you have to have a coordinator for that (TRaC-K).” [HCP03, subspecialist, ACH] |
|  | Education for potential TRaC-K users | “I think education is key. If the parents and the family members and the physicians here are all educated properly on how this is to be used and what it’s to be used for, I don’t think you’ll have a lot of, I don’t think there will be a lot of backlash....” [Family02] |

^a^ACH: Alberta Children’s Hospital.

^b^MHRH: Medicine Hat Regional Hospital.

^c^TRaC-K: Telemedicine Rounding and Consultation for Kids.
